# Acoustic metamaterials-driven transdermal drug delivery for rapid and on-demand management of acute disease

**DOI:** 10.1038/s41467-023-36581-2

**Published:** 2023-02-16

**Authors:** Junhua Xu, Hongwei Cai, Zhuhao Wu, Xiang Li, Chunhui Tian, Zheng Ao, Vivian C. Niu, Xiao Xiao, Lei Jiang, Marat Khodoun, Marc Rothenberg, Ken Mackie, Jun Chen, Luke P. Lee, Feng Guo

**Affiliations:** 1 Department of Intelligent Systems Engineering, Indiana University, Bloomington, IN 47405 USA; 2 Bloomington High School South, Bloomington, IN 47401 USA; 3 Department of Bioengineering, University of California, Los Angeles, Los Angeles, CA 90095 USA; 4 Division of Allergy and Immunology, Cincinnati Children’s Hospital Medical Center, University of Cincinnati, Cincinnati, OH 45229 USA; 5 Gill Center for Biomolecular Science, and Department of Psychological and Brain Sciences, Indiana University, Bloomington, IN 47405 USA; 6 Department of Medicine, Brigham and Women’s Hospital, Harvard Medical School, Boston, MA 02115 USA; 7 Department of Bioengineering, Department of Electrical Engineering and Computer Science, University of California at Berkeley, Berkeley, CA 94720 USA; 8 Institute of Quantum Biophysics, Department of Biophysics, Sungkyunkwan University, Suwon, Korea; 9 Present Address: Biopharmaceutical Research Institute, West China Hospital, Sichuan University, Chengdu, 610041 Sichuan China

**Keywords:** Biomedical engineering, Fluidics, Drug delivery, Drug delivery

## Abstract

Transdermal drug delivery provides convenient and pain-free self-administration for personalized therapy. However, challenges remain in treating acute diseases mainly due to their inability to timely administrate therapeutics and precisely regulate pharmacokinetics within a short time window. Here we report the development of active acoustic metamaterials-driven transdermal drug delivery for rapid and on-demand acute disease management. Through the integration of active acoustic metamaterials, a compact therapeutic patch is integrated for penetration of skin stratum corneum and active percutaneous transport of therapeutics with precise control of dose and rate over time. Moreover, the patch device quantitatively regulates the dosage and release kinetics of therapeutics and achieves better delivery performance in vivo than through subcutaneous injection. As a proof-of-concept application, we show our method can reverse life-threatening acute allergic reactions in a female mouse model of anaphylaxis via a multi-burst delivery of epinephrine, showing better efficacy than a fixed dosage injection of epinephrine, which is the current gold standard ‘self-injectable epinephrine’ strategy. This innovative method may provide a promising means to manage acute disease for personalized medicine.

## Abstract (web summary)

Treating acute disease like anaphylaxis is challenging due to the inability to administer therapeutics in a timely manner and regulate pharmacokinetics precisely within a short time window. Here the authors develop active acoustic metamaterials-driven transdermal drug delivery for rapid and on-demand acute disease management.

## Introduction

Acute disease comes on rapidly, lasts a short time, causes distinct symptoms, and sometimes even threatens an individual’s life. For example, anaphylaxis is a severe, unpredictable, and life-threatening allergic reaction affecting millions worldwide^1,2^. An anaphylactic reaction may cause cardiovascular collapse, severe laryngeal edema, bronchospasm, rapid loss of consciousness, and even death, all within minutes^3^. A medical device to inject a fixed dosage of epinephrine, “self-injectable epinephrine” or “Epi-pen”, is the gold standard for treating anaphylaxis. However, the ineffective rate of Epi-pen is up to 30%^4^, partly related to delayed and/or low/high dosage drug administration. Because of differences in patients’ age, weight, gender, and other metrics, different patients react differently to the same amount of epinephrine^5^. Even the same patient may require different amounts of epinephrine in different onsets. Thus, there is a tremendous need to advance current personalized therapy for the dynamic administration of therapeutics of a customized dosage within a specific therapeutic time window, especially for treating acute diseases that may have sudden onset of symptoms in real-world settings^6,7^.

Transdermal delivery has emerged as an attractive alternative solution for personalized therapy^8^. Compared to oral delivery and parenteral injections, the transdermal delivery method has several advantages, including its minimally invasive nature, pain-free operation, high-patient compliance, and bypass of gastrointestinal or liver metabolism^9^. So far, several methods have been developed for the transdermal delivery of small molecules and macromolecules up to tens of kilodaltons for various clinical applications. Topical formulations^10^, chemical enhancers^11^, and iontophoresis^12^ can drive small lipophilic drugs into the stratum corneum and slowly release low-dose drugs into the viable epidermis over hours. Moreover, transdermal delivery systems targeting stratum corneum have been developed using microneedles^13,14^, thermal ablation^15^, microdermabrasion^16^, and electroporation^17^. However, current transdermal delivery is still not ready for personalized therapy to treat acute disease unless it can fulfill the following criteria: (1) excellent skin permeability within a short time; (2) timely release of therapeutics with customized dosage; (3) deeper tissue transport; and (4) convenient and/or automated administration.

Ultrasound has been intensively used for transdermal drug delivery due to its several advantages, including noninvasiveness, portability, cost-effectiveness, and digital regulation^18^. Ultrasound has increased skin permeability without damaging deeper tissues by disrupting the stratum corneum using oscillating cavitational bubbles or low-frequency sonophoresis^19,20^. Although the FDA has approved handheld sonophoresis devices,^21^ challenges remain to use current ultrasound methods for personalized therapy in treating acute disease due to lengthy sonication, slow percutaneous transport, and/or insufficient dosage control. Acoustic metamaterials manipulate and control sound waves at subwavelength scales, using artificially designed structures to break through the regulation of sound waves by conventional materials in nature^22–24^. Together with digital regulation, we develop active acoustic metamaterials for the temporospatial regulation of local sound waves and streaming fields to improve transdermal drug delivery.

**Fig. 1:**
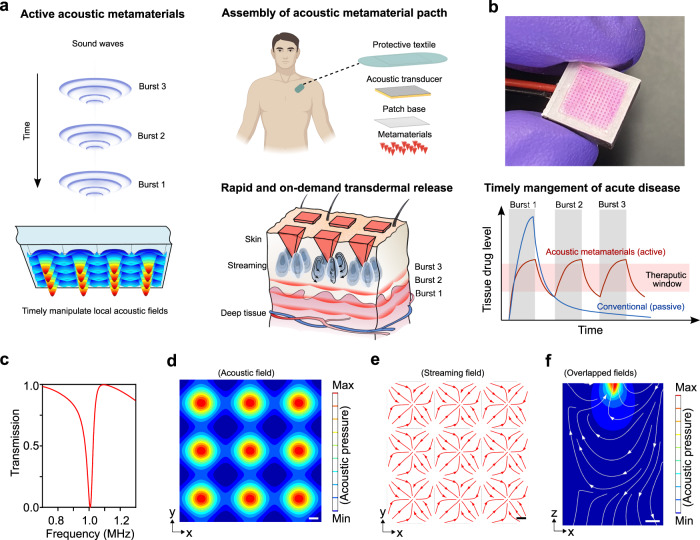
Rapid and on-demand transdermal drug delivery driven by active acoustic metamaterials for timely managing acute disease. **a** Schematics of the rapid and programmable transdermal drug delivery for timely controlling pharmacokinetics and managing the acute disease by an active acoustic metamaterial patch. **b** A presentative image of an acoustic metamaterial patch. **c** The calculated transmission curve of the designed acoustic metamaterials. Top view of the simulated acoustic field (**d**) and acoustic streaming field (**e**) above the metamaterial structures. **f** Sideview of overlapped simulated acoustic and streaming fields above one metamaterial structure.

Here, we innovate active acoustic metamaterials (Fig. 1a) to achieve convenient and minimally invasive transdermal drug delivery for rapid and on-demand acute disease management via the precise and timely control of pharmacokinetics. We integrate active acoustic metamaterials into a compact therapeutic path device that (1) increases skin permeability by creating micron-scale pathways into the skin via sharp metamaterial structures, (2) actively drives drugs through porous metamaterial structures into the superficial dermis and deep tissues via localized acoustic streaming, and (3) enables the dynamic delivery when combined with digital regulation. Using an animal model of anaphylaxis, we demonstrate that the acoustic metamaterial patch effectively rescues mice from anaphylaxis via a multi-burst release of epinephrine, showing better efficacy than the current “self-injectable epinephrine” strategy as a fixed-dose needle injection of epinephrine. This technology provides exciting perspectives for percutaneous transport, highlighting its potential in personalized therapy of acute disease.

## Results

### Device design and working principle

**Fig. 2:**
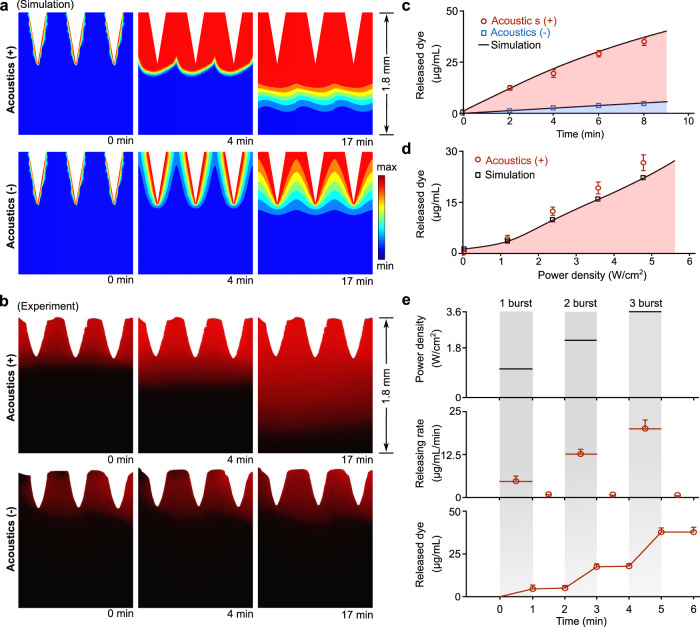
Programmable delivery in vitro. **a** Simulation results describe the dye release dynamics via acoustic metamaterials. Acoustics (+) and Acoustics (−) indicate the acoustic metamaterials-mediated treatments with and without acoustic stimulation. **b** Corresponding experimental results show the side view of RhB dye release dynamics in gels. **c** Quantification of experimental (Acoustics (+), red circles; Acoustics (−), blue squares) and simulated (black curves) accumulative RhB dye release over time (mean ± s.e.m., *n* = 3 independent experiments). **d** Dependence of experimental (red circles) and simulated (black squares) dye release on acoustic power density via acoustic metamaterials (mean ± s.e.m., *n* = 3 independent experiments). **e** Programable delivery of RhB dye with controlled release rate and dose overtime via a multi-burst stimulation (mean ± s.e.m., *n* = 3 independent experiments).

The patch consists of a piezoelectric transducer and acoustic metamaterials as a plate with periodically distributed sharp metamaterial structures (Fig. 1b). The metamaterial structures loaded with therapeutics penetrate the human epidermis. By applying resonant radio frequency (RF) signals, the acoustic metamaterial patch could be excited to generate a localized acoustic field and acoustic streaming around the metamaterial structures. The localized streaming may drive and transport therapeutics from porous sharp metamaterial structures into deeper tissues. By digitally regulating the RF signal parameters, such as power density and treatment time (e.g., timepoint, duration of each burst), the acoustic metamaterial patch could precisely release therapeutics into deeper skin tissues at a controlled dose and rate (Fig. 1a). Next, we designed and fabricated acoustic metamaterial patches to penetrate stratum corneum and percutaneous transport. Considering the typical thickness of the epidermis is around 100–400 μm, the patch was designed as a plate with a layer of pyramidal structures (height: 600 μm × length: 200 μm × width: 200 μm) in a square lattice distribution (Fig. S1a). We calculated the acoustic transmission through the metamaterials over frequency (Fig. 1c) and identified the resonant frequency of the designed metamaterials as around 1.06 Mhz. With the resonant frequency and metamaterial design, we simulated the 3D distributions of the acoustic field and acoustic streaming field (Fig. S2). The designed metamaterials split an acoustic beam into multiple localized sub-wavelength acoustic spots around the pyramidal structures (Fig. 1d) and induced localized acoustic streaming (Fig. 1e). Using the simulation models, we studied the acoustic streaming caused by acoustic metamaterials. We found four 3D streaming vortices formed around a sharp pyramidal structure. Theoretical and experimental results both showed that streaming lines flowed down from the pointed tip toward the base of the pyramid structure, then rotated up and flowed toward the upper space (simulation, Supplementary Movie 1; experiment, Supplementary Movie 2), indicating acoustic streaming may contribute as a driving force to transport therapeutics from the pyramid’s surface toward deeper tissues. We further simulated the distributions of the acoustic field and acoustic streaming above these pyramid tips (Fig. 1f). We found that the 3D distribution of released dye (Fig. 2e) correlated with the streaming distribution, indicating the dominant effect of streaming in therapeutic transport. Moreover, our simulation results show that the periodicity of metamaterial structures induces local acoustic fields and further significantly increases the acoustic streaming velocity (Fig. S2d, e), highlighting the contribution of acoustic metamaterials and their power in enhancing transdermal delivery. Thus, we demonstrate a new mechanism of transdermal delivery via acoustic metamaterials.

### Programmable delivery in vitro

We tested and validated the acoustic metamaterial patch-mediated programmable drug release in vitro using theoretical and experimental approaches. We fabricated the poly (ethylene glycol) diacrylate (PEGDA) based acoustic metamaterial patches with only sharp pyramid tips loaded with rhodamine B (RhB) dyes (Fig. S1b). Due to its stability and robust fluorescence, the RhB dye was used to model the molecular drugs in vitro, ex vivo, and in vivo.^25^ We used agar gel phantoms to test drug delivery due to their similar mechano-acoustic properties to human skins.^26,27^ We predicted the dynamic processes of dye release into the agar gel from the sharp metamaterial structures with acoustic stimulation (Acoustics (+)) and without acoustic stimulation (Acoustics (−) as passive release) (Fig. 2a and Supplementary Movie 3). Then, we also conducted experiments to track the same dye release process (Fig. 2b and Supplementary Movie 4), which matches well with the simulation. Simulated and experimental results showed that the acoustic materials significantly enhanced dye release compared to passive release over the same time interval. Then, we also quantified the dye release dynamics via acoustic metamaterials with and without acoustic stimulation. Our experimental results (Fig. 2c) demonstrated that the released dye via Acoustics (+) (red circles) was significantly greater than that via Acoustics (−) (blue squares), which matches well with our theoretical prediction (black curves). Moreover, we also investigated the dependence of dye release on acoustic power density with the same treatment time (Fig. 2d). Simulation and experimental results indicated that we could modulate the dye release rate by tuning power density. Furthermore, to test the programmable delivery, we quantified the rate and accumulative dose of released dye after applying a multi-burst simulation over time (1st burst, 1.2 W/cm^2^ for 1 min; 2nd burst, 2.4 W/cm^2^ for 1 min; and 3rd burst, 3.6 W/cm^2^ for 1 min) (Fig. 2e). We found that our acoustic metamaterial patch can precisely regulate the rate and dose of dye release over time, highlighting its potential for on-demand drug delivery with tight control of pharmacokinetics.

### Transdermal delivery ex vivo

**Fig. 3:**
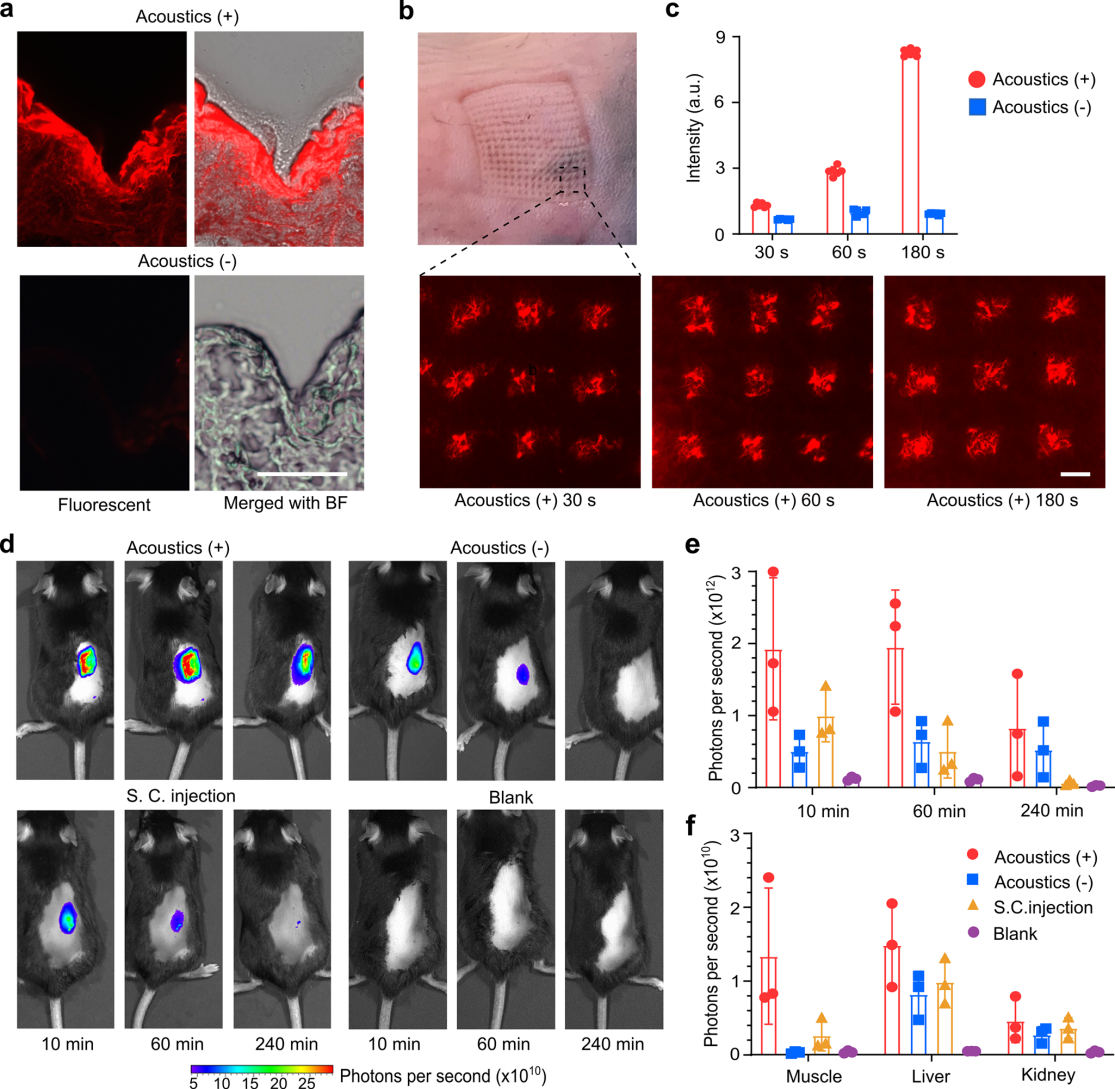
Transdermal delivery ex vivo and in vivo. **a** Histological images of mouse skin tissues after the RhB dye release treatments via acoustic metamaterials. Acoustics (+) and Acoustics (−) indicate the acoustic metamaterials-mediated treatments with and without acoustic stimulation. The experiments were repeated three times independently with similar results. **b** After acoustic metamaterial treatments, representative images of mouse skin indicated acoustic-mediated RhB dye delivery for 30, 60, or 180 s (fluorescent images). **c** After acoustic metamaterials-mediated delivery, quantification of ex vivo RhB dye release over time (mean ± s.e.m., *n* =  6 independent experiments). **d** IVISR images of mice post dye delivery (10, 60, or 240 min) via acoustic metamaterials (Acoustics (+) or Acoustics (−)) and subcutaneous injection (S.C. injection) methods, respectively (red/blue indicates high/low photon counts). **e** Quantification of in vivo RhB dye release using the different methods (mean ± s.e.m., *n* = 3 mice per group, independent experiments). **f** Presence of RhB dye in mouse muscle, liver, and kidney 24 h after the indicated treatments (mean ± s.e.m., *n* = 3 mice per group, independent experiments). Scale bar, 200 µm.

We validated the acoustic metamaterial patches using fresh mouse skin tissues to evaluate stratum corneum penetration and control drug release. Based on the excellent mechanical property of the acoustic metamaterial patch (Fig. S3), we applied the RhB dye-loaded patch device to the freshly harvested mouse skin. After applying acoustic waves for 3 min with a power density of 2.4 W/cm^2^, we characterized the dye release within the histologically sectioned mouse skin tissues using fluorescent imaging. As shown in Fig. 3a, our patch device penetrated the mouse skin with a maximal penetration depth of ~500 μm and released dye into the epidermis and deeper tissues with a maximal diffusion depth of ~1000 μm highlighting its ability for transdermal delivery in vivo. Moreover, histological section images also showed deeper tissue transport and a higher dose of released dye surrounding the pyramidal structure after the patch treatment with acoustic stimulation (acoustics (+)) compared to the same treatment condition without acoustic treatment (acoustics (−) or passive diffusion). Therefore, these results clearly showed that (1) the metamaterial structures penetrate mouse skin, and (2) acoustics actively releases the dye inside mouse skin tissues and significantly enhances percutaneous transport compared to the passive dye diffusion. Furthermore, to achieve transdermal drug delivery with precise dose control, we explored the ability of the patch devices to quantitatively release dye into mouse skin by tuning acoustic treatment (e.g., adjusting treatment time while maintaining the power density at 2.4 W/cm^2^). After applying the RhB dye-loaded patch device to the fresh mouse skin tissues, we observed transdermal dye release under different treatment times (e.g., 30, 60, and 180 s in Fig. 3b). We also quantified the released dye over additional treatment times (Fig. 3c). We found that the patch device could control the dose of transdermal RhB by tuning acoustic treatment time. The acoustic metamaterial patch (Acoustics (+)) released about 9.3 times as much transdermal RhB dye as released passively (Acoustics (−)) during the same treatment time. Thus, we demonstrated that the acoustic metamaterial patch could achieve transdermal drug delivery with precise drug dose and release rate control.

### Transdermal delivery in vivo and biodistribution

We tested the acoustic metamaterials-meditated transdermal delivery in vivo using live C57BL/6 mice and compared the delivery efficiency with subcutaneous injection (S.C. injection). We conducted the transdermal delivery of RhB dye with the same loading dose to the mice via the acoustic metamaterial patch with (Acoustics (+)) or without acoustic stimulation (Acoustics (−)) and S.C. injection methods, respectively. After applying the different techniques, the transdermal delivered RhB dyes will diffuse away from the administration area within the mouse skin tissue and be taken up by the circulatory system via systemic delivery. We visualized and analyzed the dynamic diffusion and absorption process of transdermally delivered dyes within each living animal using an In Vivo Imaging Systems (IVIS) system, where the photon counts represent the intensity of RhB dyes. The IVIS images of a representative mouse from four different treatment groups (Acoustics (+), Acoustics (−), S.C. injection, and blank) were taken at pre-determined time intervals (10, 60, or 240 min in Fig. 3d). The IVISR images showed that the mouse treated by the Acoustics (+) treatment showed higher photon intensity, a larger area of diffused dye and that the dye was retained for a longer time within the skin tissue than the Acoustics (−) or S.C. injection treatment groups. In contrast, the mouse without treatment (blank) showed the lowest photon intensity. Moreover, we also quantified these dynamic processes by counting the average photon intensity over time in the different treatment groups (mean ± s.e.m., *n* =  3 mice per group, independent experiments, Fig. 3e). The results also showed that the Acoustics (+) group had a remarkedly higher dose of transdermally delivered dyes within 1 h post the transdermal delivery compared to the Acoustics (−) (passive diffusion) or S.C. injection treatment groups, which is possibly caused by the acoustically enhanced dye diffusion within the superficial dermis and deeper tissues.

To further explore the diffusion and uptake of transdermal delivery in vivo, we investigated the biodistribution of transdermally delivered dyes within different treatment groups (Acoustics (+), Acoustics (−), S.C. injection, and Blank). Twenty-four hours after transdermal delivery, we harvested and imaged mouse organs (e.g., muscle, liver, and kidney) from the different treatment groups using the IVISR system (Fig. S4). We quantified the averaged photon intensity of the three organs from each treatment group (mean ± s.e.m., *n* =  3 mice per group, independent experiments, Fig. 3f). The results showed that the dosage of RhB dyes in muscle tissues of the Acoustics (+) group was significantly greater than that in the Acoustics (−) group (or S.C. injection group). The reason may be that the acoustic waves enhanced the diffusion and uptake of transdermally delivered dyes into subcutaneous blood vessels, which further transferred them from the administration site into distal muscle tissues. Similarly, the dose of RhB dyes in the liver and kidney tissues from the Acoustic (+) group was slightly higher than the Acoustics (−) or S.C. injection group. Taken together, the acoustic metamaterials-mediated transdermal delivery method exhibited rapid and enhanced transdermal delivery in vivo, enabling a better means than the S.C. injection and passive diffusion methods.

### Treating anaphylaxis through dynamic transdermal delivery of epinephrine

**Fig. 4:**
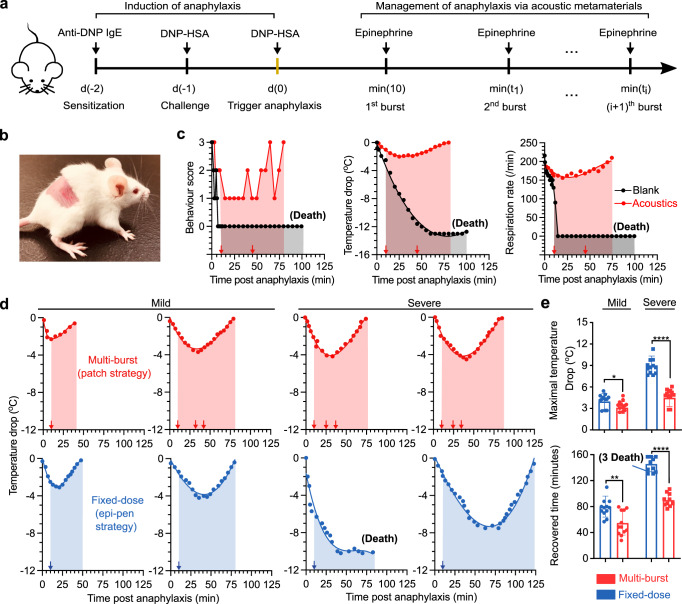
Treating anaphylaxis via on-demand delivery of epinephrine. **a** Schematic illustrates the induction of anaphylaxis in mice and management of anaphylaxis using acoustic metamaterials-mediated transdermal epinephrine delivery. **b** Images of a mouse after the acoustic metamaterials-mediated delivery. **c** The behavior score, body temperature, and respiratory rate of mice during anaphylaxis with epinephrine treatment via acoustic metamaterials (Acoustics) or without treatment (Blank). **d** The dynamic body temperature change of mice with mild (left) or severe (right) anaphylactic reactions during a multi-burst delivery of epinephrine via acoustic metamaterial patches and a fixed dosage needle injection of epinephrine. The arrows indicate the time of epinephrine injection. **e** The maximal temperature drops and recovery time of mice with mild or severe anaphylaxis during the epinephrine treatments via multi-burst or fixed-dose deliveries (mean ± s.e.m., *n* = 12 mice per group, independent experiments).

As a proof-of-concept, we evaluated the management of acute and heterogeneous anaphylaxis events through the dynamic transdermal delivery of epinephrine via the acoustic metamaterial patch device. We established a mouse model of anaphylaxis using a passive systemic anaphylaxis protocol (details in methods). We then tested the on-demand delivery of epinephrine through the acoustic metamaterial patch for treating anaphylaxis (Fig. 4a). About 10 min post-induction of anaphylaxis, the anaphylactic mice received the first burst of epinephrine (2.5 μg). If needed, the anaphylactic mouse received a second burst (or multiple bursts) of epinephrine (2.5 μg per burst) until anaphylaxis was reversed. Before conducting the epinephrine delivery for anaphylaxis treatment in vivo, we fabricated the acoustic metamaterial patches with only the pyramidal structures loaded with epinephrine and tested the controllable delivery of epinephrine in vitro (Fig. S5). We demonstrated that the single patch device could precisely deliver epinephrine with the desired dose by applying one or more bursts over time. The device can be used on freely-moving mice in a minimally invasive manner (Fig. 4b and Supplementary Movie 5).

To validate the efficacy of our acoustic metamaterial patch in treating acute anaphylaxis, we tracked the dynamic progression and treatment response of anaphylactic mice using body temperature, respiration, behavior score, and serum histamine level (Fig. S6). Right after the injection of the allergen DNP–HSA, the behavior score, body temperature, and respiration of three mice dropped dramatically, indicating the successful induction of anaphylaxis (Fig. 4c). The anaphylactic mouse received a multi-burst delivery of epinephrine via the acoustic metamaterial patch (red squares and red arrows show the first burst 10 min post anaphylaxis and the second burst 45 min post anaphylaxis). The reversal of anaphylaxis was demonstrated by the normalized behavior score, body temperature, and respiration. In contrast, anaphylactic mice without any treatment (black triangles) died. These results indicated that the multi-burst epinephrine delivery via the acoustic metamaterial patch could effectively treat acute anaphylaxis.

Compared with the current “self-injectable epinephrine” strategy that relies on the needle injection of a fixed dosage of epinephrine, the acoustic metamaterial patch could precisely adjust the dose of epinephrine administered via dynamic delivery, highlighting its potential for personalizing the treatment of acute anaphylaxis of varying severity. To mimic anaphylaxis of different severity (e.g., mild and severe conditions), we induced anaphylaxis in mice of different severity by modifying the allergen dosage. After triggering variable anaphylaxis responses in mice (12 for mild; 12 for severe), we tested and compared the acoustic metamaterial patch method (a multi-burst delivery of epinephrine with 2.5 μg per burst) with the classic “self-injectable epinephrine” strategy (fixed-dose S. C. injection of 5 μg epinephrine). The representative body temperature curves (Fig. 4d, find the whole dataset in Fig. S7) showed that the acoustic metamaterial patch method effectively rescued the mice with mild and severe anaphylactic reactions via a multi-burst delivery of epinephrine (in red). In contrast, the “self-injectable epinephrine” strategy only succeeded in routinely saving mice with mild anaphylactic reactions but failed to rescue 25% of the mice with severe anaphylactic reactions (in green). Moreover, we also found that compared to the “self-injectable epinephrine” strategy, the acoustic metamaterial patch significantly reduced the maximal drop in body temperature (>4 °C) and shortened the average recovered time (>35 min) of mice with severe conditions (Fig. 4e). Moreover, the acoustic metamaterial patch method showed a slight, but significantly better performance than the fixed-dose injection of epinephrine for mice with severe anaphylaxis. Thus, we demonstrated that the acoustic metamaterial patch provides an alternative, convenient, and effective means for treating an acute disease of varying severity, such as anaphylaxis, highlighting its potential for personalized therapy.

## Discussion

We have demonstrated a new class of acoustic metamaterial patch devices that offers active, dynamic, minimally invasive, and convenient transdermal delivery of therapeutics into deep skin tissue, providing a digital percutaneous transport method for precisely regulating pharmacokinetics. This device exploits acoustic metamaterial innovation, therapeutic patch integration, and digital regulation strategies to achieve both state-of-the-art functions and a multi-burst of epinephrine that allows the reversal of mild and severe acute anaphylaxis. The patch device can be adapted for transdermal delivery of a range of therapeutics with excellent dose control over time and clear clinical implications.

The performance and functionality of the first-generation patch devices can be improved. The drug release rate could be optimized via the design and fabrication of metamaterial structures, selection of materials, and drug encapsulation, as required for specific personalized therapies. Additionally, the material and treatment condition of the acoustic metamaterial patch device is biocompatible. To improve the safety of the patch administration, a digital patch holder could be incorporated to penetrate the patch pyramids into skin tissue only when needed and to remove it right after epinephrine administration and thus keep the patch away from skin tissue when not needed. Furthermore, integrating digital control, wireless communications, and power sources in a compact and lightweight format would significantly enhance device portability and wearability. A closed-loop control system could also be implemented to receive triggering signals from sensors for real-time treatment of acute disease.

## Methods

### Simulation of acoustic metamaterials-mediated dye release

The acoustic metamaterials-mediated dye release in the agar gel was simulated using our well-developed model^28–30^ via a COMSOL Multiphysics numerical simulation software (COMSOL 5.3a, COMSOL Inc., Burlington, MA USA). The details of the simulation process are described in the Supplementary Methods.

### Fabrication of acoustic metamaterial patches

The acoustic metamaterial device was fabricated using poly (ethylene glycol) diacrylate (PEGDA) material and polydimethylsiloxane (PDMS) molds (Micro-point Ltd., Singapore). Using our well-developed fabrication procedure^31^, the device was fabricated with only periodically distributed pyramidal structures loaded with rhodamine B (RhB, Sigma-Aldrich, MO) or epinephrine (Sigma-Aldrich, MO) to precisely control drug release with the following steps. Solution 1 was prepared by mixing 0.89 ml of PEGDA (MW 700, Sigma-Aldrich, MO), 10 mg of Irgacure 2959 dissolved in 10 μl of DMSO, and 0.1 ml of 25 mg/ml RhB solution. Solution 2 was prepared by well-mixing 0.99 ml of PEGDA and 10 mg of Irgacure 2959 dissolved in 10 μl of DMSO. Then, solution 1 was loaded into the PDMS mold and treated within a vacuum for 5 min to fill the solution into the pyramid reservoirs. Then, solution 1 was wiped away from the surface of the PDMS mold. Solution 2 was immediately added to the PDMS mold and degassed for 5 min. Then, the PDMS mold was covered with a piezoelectric transducer (PZT, Jiangsu Paizhou Electronic Technology, China) and exposed to UV light (365 nm, 12 mW/cm^2^) for 1.5 min. Finally, the device with acoustic material structures was carefully removed from the mold. The morphology of the periodically distributed pyramid structures was characterized using a Leica stereomicroscope (M205FA, Germany).

### Activation of acoustic metamaterial patch devices

The acoustic metamaterial patch was driven by programmable sine radio frequency (RF) signals (frequency, 1 Mhz; duty cycle, 15%; energy density, 0–5.8 w/cm^2^; time, 0–10 min) to achieve the on-demand transdermal delivery. The programmable sine RF signals were generated by a function generator (TGP3152, Aim TTi, UK) and amplified by a power amplifier (LZY-22+, Mini-circuit, USA). After applying the programmable RF signals, the piezoelectric transducer was excited to generate acoustic waves. The generated acoustic waves were propagated through a patch base layer and interacted with the periodic structures of the designed metamaterials. Through manipulation by the acoustic metamaterials, the local acoustic fields were formed surrounding the periodic metamaterial structures and further induced local acoustic streaming surrounding the periodic metamaterial structures for actively releasing drugs from the sharp metamaterial structures into the tissues or phantoms.

### In vitro tests of digital delivery

We characterized the drug release performance after removing the RhB dye (or epinephrine) on the surfaces of acoustic metamaterials. We washed the material surfaces by inserting RhB dye (or epinephrine) loaded structures into an agar gel (length: 1 cm, width: 1 cm, thickness: 1 mm) for 30 s (without acoustic treatment). The washed devices were inserted into a piece of agar gel (length: 1 cm, width: 1 cm, thickness: 1 mm), followed by the acoustic treatment (frequency, 1 Mhz; duty cycle, 15%; energy density, 0–5.8 w/cm^2^; time, 0–10 min), and removed from the gel. The RhB dye (or epinephrine) released gels were imaged by an IX83 microscope (Olympus, Japan). The RhB distribution in the agar gel was quantified using ImageJ (NIH) by analyzing the pixel intensity. The released RhB dye was calculated based on a standard fluorescence curve and plotted using Origin 9.1 and Graph Pad Prism 9.

### Animals

All experiments were conducted following the animal guidelines of the institutional animal care and use committee (IACUC) of Indiana University as approved under protocol # 19-006. Female C57BL/6J mice (6–12 weeks of age, Envigo) weighing 18–22 g were used for testing ex vivo patch release, and female BALB/c mice (6–12 weeks of age, Envigo) weighing 18–22 g were used to develop the anaphylaxis models. Mice were housed as five mice in a cage under conventional conditions (12 h light/dark cycle at 22 °C).

### Ex vivo drug release test

The prepared RhB dye (or epinephrine) loaded acoustic metamaterial patches were inserted into freshly harvested skin tissues from BALB/c mice that had hair removed. The treatment condition (Acoustics (+): frequency, 1 Mhz; duty cycle, 15%; energy density, 2.4 w/cm^2^) was applied to three mouse skin tissues, while there was no acoustic stimulation for passive diffusion-based treatments (Acoustics (−)). After treatments, the mouse skin tissues with three different treatment times (e.g., 30, 60, or 180 s) were imaged by an IX83 microscope (Olympus, Japan). The RhB distribution in the skin was quantified using ImageJ (National Institute of Health) by analyzing the pixel intensity.

### Cryo-section of mouse skin ex vivo after dye delivery via acoustic metamaterials

Dye release ex vivo by acoustic metamaterials with (Acoustics (+)) or without acoustic stimulation (Acoustics (−)) was characterized by determining the fluorescent intensity of cryo-sectioned skin slides. First, the RhB-loaded device was fabricated by the processes above, and mouse skin was isolated from BALB/c mice. The fur was removed using the Nair® hair removal lotion (Church & Dwight Canada Corp., Canada). Two RhB-loaded devices were applied to mouse skin respectively. For Acoustics (−) treatment, the device was inserted into mouse skin for 2 min without acoustic stimulation. For Acoustics (+) treatment, the device was inserted into mouse skin for 2 min with acoustic stimulation (2.4 w/cm^2^). After treatments via acoustic metamaterials, mouse skin was immediately frozen in OCT using a tissue mold at dry ice for 1 h. The tissue sections (20 μm) were cut on a cryostat at −20 °C and were collected on glass slides for further fluorescence imaging.

### In vivo test of patch release and biodistribution

Twelve mice (C57BL/6J) were used to compare in vivo acoustic patch-enabled dynamic release to subcutaneous injection, passive release, and blank control using an in vivo imaging system (IVIS Spectrum, Perkin Elmer). The fur on the back of the mice was removed by Nair® hair removal lotion 1 day before the experiment. The mice were anesthetized and administrated RhB dye in four different groups, including (1) Acoustics (+) group (frequency, 1 Mhz; duty cycle, 15%; energy density, 2.4 w/cm^2^; treatment time, 3 min; with 4.5 μg dye per patch); (2) Acoustics (−) group (treatment time, 3 min; with 4.5 μg dye per patch); (3) subcutaneous injection (20 μl of 225 μg/ml RhB); and (4) blank group (without any treatment). After washing the skin surface three times with PBS-soaked cotton balls, the mice were imaged at three pre-determined time points (10, 60, and 240 min). At 24 h, the mice were euthanized, and the organs (e.g., liver, kidney muscle, and skin) were dissected and visualized by the IVIS imaging system to study the biodistribution of dye RhB (Fig. S3).

### Mouse model of anaphylaxis

A passive systemic anaphylaxis (PSA) model was established following reported protocols^32^. The mice (BALB/c) were sensitized by the intraperitoneal injection of 200 μl of IgE solution (as 100 μg/ml of monoclonal mouse anti-DNP IgE antibody (D8406, Clone SPE-7, Sigma-Aldrich, MO) in 0.9 % NaCl) so that the mice will receive a total of 20 μg of IgE anti-DNP. After 24 h, the sensitized mice were challenged through the intravenous injection with 100 μl of DNP–HSA solution (10 mg/ml of DNP–HSA in 0.9 % NaCl). A 100 μl of DNP–HSA solution was injected intraperitoneally into the challenged mice to trigger the anaphylactic reaction. The whole anaphylactic process lasted for about 120 min, and all mice were sacrificed after the experimental procedures (120 min).

### Characterization of anaphylaxis in mice

The mice were characterized via physiological parameters and symptoms, including rectal temperature, movement score, respiratory rate, or blood histamine level. Rectal temperature was measured using a rectal probe every 2 min in real-time. Respiratory rate was measured by counting breaths every 20 s and observing thoracic movement. Movement score was tested as follows: react actively after provoking (score 3); slow in response after initiating (score 2); no active response after starting (score 1); dead or no response at all (score 0) (Fig. S6). Blood histamine level was measured by testing whole blood samples obtained from the tail vein and quantified by an ELISA kit (Catalog No: IT6088, Geno Technology Inc., MO) (Fig. S6). Samples were all stored at −80 °C for later assessment of the concentration of histamine by an ELISA kit.

### Anaphylaxis reversal by epinephrine

The anaphylactic mice were rescued by administrating epinephrine via needle injection or acoustic metamaterials-mediated delivery. The epinephrine dosage is based on a standard of 0.01 mg/kg/dose. To explore further human application, the acoustic metamaterial patch device could be optimized to load more epinephrine and/or increase metamaterial structures. Epinephrine (Sigma-Aldrich, MO) was dissolved into 0.9% saline to reach an 80 μg/ml concentration. The epinephrine solution was loaded in a syringe ((27 G needles; 1 ml Syringes, BD) and then injected into the mice to mimic the “self-injectable epinephrine” strategy. The epinephrine-loaded acoustic metamaterial patch was inserted into the back of a mouse with removed hair and treated using different treatment conditions.

### Statistical analysis

The statistics comparing two sample groups were conducted using the Student’s *t* test. Statistical significance was denoted as following: *p < 0.05, **p < 0.01, ***p < 0.005. ****p < 0.001.

### Reporting summary

Further information on research design is available in the Nature Portfolio Reporting Summary linked to this article.

## Supplementary information

Supplementary Information

Description of Additional Supplementary Files

Supplementary Movie 1

Supplementary Movie 2

Supplementary Movie 3

Supplementary Movie 4

Supplementary Movie 5

Reporting Summary

## Source data

Source Data

## Data Availability

All relevant data supporting the key findings of this study are available within the article and its Supplementary Information files or from the corresponding author upon reasonable request. Source data are provided with this paper.
