# Author Correction: Acoustic metamaterials-driven transdermal drug delivery for rapid and on-demand management of acute disease

**DOI:** 10.1038/s41467-026-74309-0

**Published:** 2026-06-23

**Authors:** Junhua Xu, Hongwei Cai, Zhuhao Wu, Xiang Li, Chunhui Tian, Zheng Ao, Vivian C. Niu, Xiao Xiao, Lei Jiang, Marat Khodoun, Marc Rothenberg, Ken Mackie, Jun Chen, Luke P. Lee, Feng Guo

**Affiliations:** 1 Department of Intelligent Systems Engineering, Indiana University, Bloomington, IN 47405 USA; 2 Bloomington High School South, Bloomington, IN 47401 USA; 3 Department of Bioengineering, University of California, Los Angeles, Los Angeles, CA 90095 USA; 4 Division of Allergy and Immunology, Cincinnati Children’s Hospital Medical Center, University of Cincinnati, Cincinnati, OH 45229 USA; 5 Gill Center for Biomolecular Science, and Department of Psychological and Brain Sciences, Indiana University, Bloomington, IN 47405 USA; 6 Department of Medicine, Brigham and Women’s Hospital, Harvard Medical School, Boston, MA 02115 USA; 7 Department of Bioengineering, Department of Electrical Engineering and Computer Science, University of California at Berkeley, Berkeley, CA 94720 USA; 8 Institute of Quantum Biophysics, Department of Biophysics, Sungkyunkwan University, Suwon, Korea; 9 Present Address: Biopharmaceutical Research Institute, West China Hospital, Sichuan University, Chengdu, 610041 Sichuan China

**Keywords:** Biomedical engineering, Fluidics, Drug delivery, Drug delivery

Correction to: *Nature Communications* 10.1038/s41467-023-36581-2, published online 16 February 2023

In the version of this article initially published, there was an error in Fig. 3d where the acoustics (+); 60 min mouse image was an inadvertent duplicate of the acoustics (+); 10 min mouse image to its left, while in the Fig. 3e acoustics (–); 10 min column, an extraneous data point appearing between 1 and 2 on the *y*-axis has been removed. The figure is amended in the HTML and PDF versions of the article.

