# Supplementary material for: Acoustic metamaterials-driven transdermal drug delivery for rapid and on-demand management of acute disease
Source: Nat Commun. 2023 Feb 16;14:869. doi: 10.1038/s41467-023-36581-2 (PMC9935629; doi:10.1038/s41467-023-36581-2)
Supplement: Supplementary file 1 — Supplementary Information [file 41467_2023_36581_MOESM1_ESM.pdf]

Supporting information:

Acoustic metamaterials-driven transdermal drug delivery for rapid and on-demand management of acute disease

Junhua Xu,[1,§,#] Hongwei Cai,[1#] Zhuhao Wu,[1] Xiang Li,[1] Chunhui Tian,[1] Zheng Ao,[1] Vivian C. Niu,[1,2] Xiao Xiao,[3] Lei Jiang,[1] Marat Khodoun,[4] Marc Rothenberg,[4] Ken Mackie,[5] Jun Chen,[3*] Luke P. Lee,[6,7,8*] and Feng Guo[1*]

1. Department of Intelligent Systems Engineering, Indiana University, Bloomington, IN 47405, United States
2. Bloomington High School South, Bloomington, IN 47401, United States
3. Department of Bioengineering, University of California, Los Angeles, Los Angeles, CA 90095, United States
4. Division of Allergy and Immunology, Cincinnati Children's Hospital Medical Center, University of Cincinnati, Cincinnati, OH 45229, United States
5. Gill Center for Biomolecular Science, and Department of Psychological and Brain Sciences, Indiana University, Bloomington, IN 47405, United States
6. Department of Medicine, Brigham and Women's Hospital, Harvard Medical School, Boston, MA 02115, United States
7. Department of Bioengineering, Department of Electrical Engineering and Computer Science, University of California at Berkeley, Berkeley, CA 94720, United States
8. Institute of Quantum Biophysics, Department of Biophysics, Sungkyunkwan University, Suwon, Korea

§ Current address: Biopharmaceutical Research Institute, West China Hospital, Sichuan University, Chengdu, Sichuan, 610041, China

# Contributed equally to this work

* Corresponding email address:

jun.chen@ucla.edu (J.C.), lplee@bwh.harvard.edu (L.P.L.), or fengguo@iu.edu (F.G.)

List of Contents

Supplementary Methods

Theoretical prediction of acoustic metamaterials mediated drug release.

Supplementary Table

Table S1. Parameter list for simulation.

Supplementary Figures

Fig.S1 Design, device, and function of the acoustic metamaterial patches.
Fig.S2 Simulation of the acoustic metamaterial patches
Fig.S3 Mechanical property of the acoustic metamaterial patches.
Fig.S4 In vivo dye distribution mediated by acoustic metamaterial patches and other methods.
Fig.S5 Controlled release of epinephrine via acoustic metamaterial patches.
Fig.S6 Characterization of a mouse model of anaphylaxis.
Fig.S7 Body temperature variation of anaphylactic mice with different treatments.

Supplementary Methods

Theoretical prediction of drug release mediated by acoustic metamaterials: The governing perturbation equations for acoustic fields in the porous media consist of a balance of linear momentum and mass. Considering the effects of the 2-phase porous metamaterial structure, the standard equation of porous medium dynamics can be written as[1]:

$$\rho\left(\frac{\partial \boldsymbol{v}}{\partial \mathrm{t}}+\boldsymbol{v}\cdot\nabla\boldsymbol{v}\right)=-\nabla p-\gamma\boldsymbol{w} \tag{1}$$

$$\frac{\partial}{\partial \mathrm{t}}(\phi\rho)+\nabla\cdot(\phi\rho\boldsymbol{v})=0 \tag{2}$$

$$\boldsymbol{w}=\phi(\boldsymbol{v}-\boldsymbol{u}) \tag{3}$$

The actual density of the fluid is ρ, the volume fraction of porous media is ϕ, the interstitial fluid velocity is $\boldsymbol{v}$, $\gamma\equiv 1/K$ is the inverse of the hydraulic conductivity K, $\boldsymbol{u}$ is the velocity of the porous solid frame, and $\boldsymbol{w}$ is the relative velocity of the fluid. Under a harmonic force, the motion of the fluid is generally not harmonic. It is generally composed of two components: (1) a first-order component of the same period as the activation force, and (2) a second-order stable component (acoustic streaming). Taking the first and second-order components into account, we can write[2]:

$$\boldsymbol{w}=\boldsymbol{w_1}+\boldsymbol{w_2} \tag{4}$$

$$\boldsymbol{v}=\boldsymbol{v_1}+\boldsymbol{v_2} \tag{5}$$

$$\boldsymbol{u}=\boldsymbol{u_1}+\boldsymbol{u_2} \tag{6}$$

$$\rho=\rho_0+\rho_1+\rho_2 \tag{7}$$

$$p=p_0+p_1+p_2 \tag{8}$$

$$\phi=\phi_0+\phi_1+\phi_2 \tag{9}$$

For the first-order equation, we assume that acoustic waves travel in a uniform media, and the fluid and porous frame move together with the same velocity. Thus, $\boldsymbol{w_1}$ and ϕ_1 vanish in the first-order equation, and we get: $\frac{\partial \rho_1}{\partial \mathrm{t}}+\rho_0(\nabla\cdot\boldsymbol{v_1})=0$

$$\rho_0\left(\frac{\partial \boldsymbol{v_1}}{\partial \mathrm{t}}+\boldsymbol{v_1}\cdot\nabla\boldsymbol{v_1}\right)=-\nabla p_1 \tag{10}$$

For the second-order equation, since there is no streaming in the porous frame, the second-order frame velocity $\boldsymbol{u_2}$ is zero. Expanding the equation to the second order and averaging the equation over a cycle yields:

$$\gamma\boldsymbol{w_2}=-\nabla p_2-\rho_0\nabla\cdot\langle v_1 v_1\rangle \tag{11}$$

$$\nabla\cdot\boldsymbol{w_2}=-\phi_0\frac{\nabla\cdot\langle\rho_1\boldsymbol{v_1}\rangle}{\rho_0} \tag{12}$$

The second-order equation has a form of Darcy's law, supplemented with a streaming force term. For the acoustically-enhanced convection-diffusion of dye and drug, the governing equation is the conventional diffusion equation[3]:

$$\frac{\partial \mathrm{c}}{\partial \mathrm{t}}=\nabla\cdot(D\nabla\mathrm{c})-\nabla(\boldsymbol{v^c}c)+\mathrm{R} \tag{13}$$

c is the species concentration, *D* is the diffusivity, $\boldsymbol{v^c}$ is the velocity field that the species is moving with. *R* describes sources or sinks of the quantity *c*.

Combining the second-order equation, we can get:

$$\nabla^2 p_{\mathbf{2}} = \nabla \cdot \left(-\rho_0 \nabla \cdot \langle \boldsymbol{v_1 v_1} \rangle + \frac{\gamma \phi_0}{\rho_0} \langle \rho_1 \boldsymbol{v_1} \rangle \right) \quad (14)$$

Thus, the streaming speed in the porous media also has the estimated relation with acoustic pressure:

$$\boldsymbol{v_{porous}} \sim \omega P^2 \quad (15)$$

This streaming results in an effective diffusivity:

$$D_{eff} \sim \omega P^2 \quad (16)$$

If we simplify the convection-diffusion to 1D diffusion from the patch to the porous media, with a constant concentration c_0 at the surface of the patch. We can get:

$$\frac{\partial \mathrm{c}}{\partial \mathrm{t}} = D \frac{\partial^2 \mathrm{c}}{\partial \mathrm{x}^2} \quad (17)$$

$$D = D_0 + D_{eff} \quad (18)$$

D_0 is the diffusivity of dye in the porous media. The regulation of this estimation equation is:

$$c = 0 \quad at \quad t = 0 \quad (19)$$

$$c = c_0 \quad at \quad t = 0 \quad (20)$$

With a solution:

$$c(x,t) = c_0 \left[1 - \mathrm{erf}\left(\frac{x}{\sqrt{4Dt}} \right) \right] \quad (21)$$

$$D = D_0 + D_{eff} \quad (22)$$

$$D_{eff} \sim \omega P^2 \quad (23)$$

Integrating along x to get d (the total released dye mass) at time t:

$$d = c_0 \sqrt{\frac{4Dt}{\pi}} \quad (24)$$

$$D = D_0 + D_{eff} \quad (25)$$

$$D_{eff} \sim \omega P^2 \quad (26)$$

To reduce the computational effort, we only solved for one pyramid in the periodic metamaterial structure in 3D, because the pyramidal structures' vibrations are periodic in both the x- and y-directions. Based on the above-discussed theoretical derivation, the numerical procedure is divided into three steps: (1) solving the acoustic field (1st-order problem) in the porous media domain; (2) solving the 2nd-order problem in a porous domain based on the 1st-order result from the first step and obtaining the streaming field; (3) solving for the diffusion of dye in porous domain assisted by acoustic streaming. COMSOL 5.3a (the COMSOL Group) was employed for the calculations following the above steps with all the parameters listed as shown in **Table S1**. A computational domain was used for simulating a unit of periodic metamaterial structures (**Fig.S2a**). In step (1), the predefined "Pressure acoustics" modified with "Poroacoustics" physics was used to calculate the acoustic field distribution (1st-order problem) in porous media. A "Periodic" boundary condition, which confines periodic connection of pyramids in the array and fluidic

domains, was applied to side the two boundaries of the porous domain. The top boundary was set as “normal impedance” equal to that of the porous media to eliminate wave reflection. An activation of defined periodicity was applied to the patch boundary to account for acoustic vibration. Based on these settings, a “Frequency Domain” solver was used to solve the abovementioned physics together at the driving frequency (1.01 MHz). In step (2), “Darcy’s law” physics was used to solve the 2nd-order problem (Acoustic streaming) in porous media. The mass and force source terms were imposed by adding “weak contribution” and “volume force” conditions, respectively. Similarly, the condition, which confines periodic connection of pyramids in the array and fluidic domain, was applied to two surrounding boundaries. And an “outlet” boundary condition, which indicates no pressure difference on the two sides of a boundary, was imposed at the media-media interfaces. This physics is solved via a “Stationary” solver using the 1st-order solution of the previously mentioned “Frequency Domain” solver. As the last step, the “Transport of Diluted Species” was used to solve the diffusion problem of the dye. A constant concentration of dye is set on the surface of the patch to account for diffusion. All the walls of porous media were set as no flux boundary conditions. These physics were then solved via a "Time-Dependent" solver in a total of 1020 seconds with an interval of 0.1s by using the 2nd-order solution of the previously mentioned “Stationary” solver.

Supplementary Table

Fluid volume	1 x 0.5 x 0.5 mm^3
Acoustic speed in fluid	1490 m s^{-1}
Fluid density	999.62 kg m^{-3}
Dynamic viscosity of water	1.0093×10^{-3} Pa s
Pressure-wave speed in metamaterials	2700 m s^{-1}
Shear-wave speed in metamaterials	1160 m s^{-1}
Density of metamaterials	1180 kg m^{-3}
Porosity of gel	0.9805
Permeability of gel	367 nm^2
Diffusion coefficient	4.5 x 10^{-6} cm^2 s^{-1}

Table S1. Parameter list for simulation.

Supplementary Figures

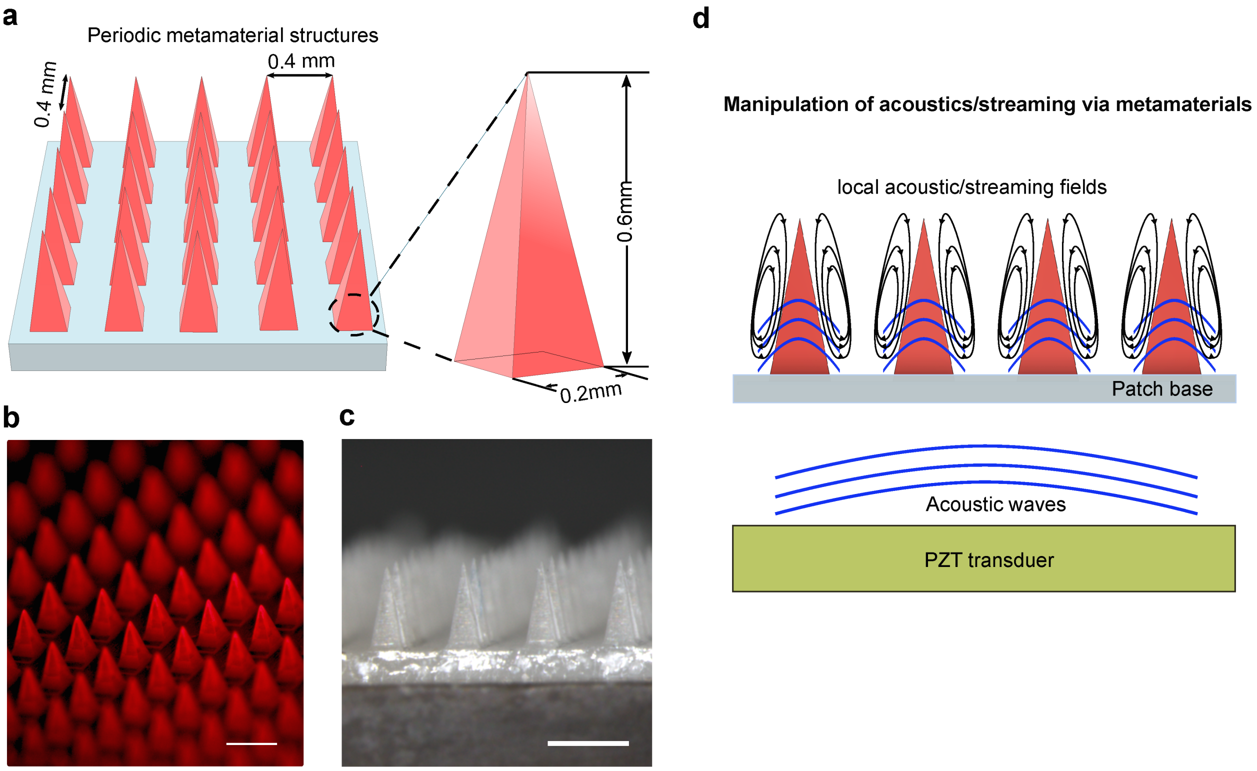

Fig.S1 Design, device, and function of the acoustic metamaterial patches. (**a**) The design of acoustic metamaterials as a plate with a layer of pyramidal structures (height:600 μm x length:200 μm x width:200 μm) in a square lattice distribution (pyramid center to pyramid center distance: 400 μm). (**b**) The fabricated acoustic metamaterial plate was loaded with only the pyramidal-shaped metamaterial structures with RhB dye. The experiment was repeated 3 times independently with similar results. (c) The fabricated acoustic metamaterial patch consists of a transducer and an acoustic metamaterial plate. The experiment was repeated 3 times independently with similar results. (d) Schematic showing the manipulation of acoustic waves and acoustic streaming via the periodic structures of the acoustic metamaterial patch device. (Scale bar: 400 μm)

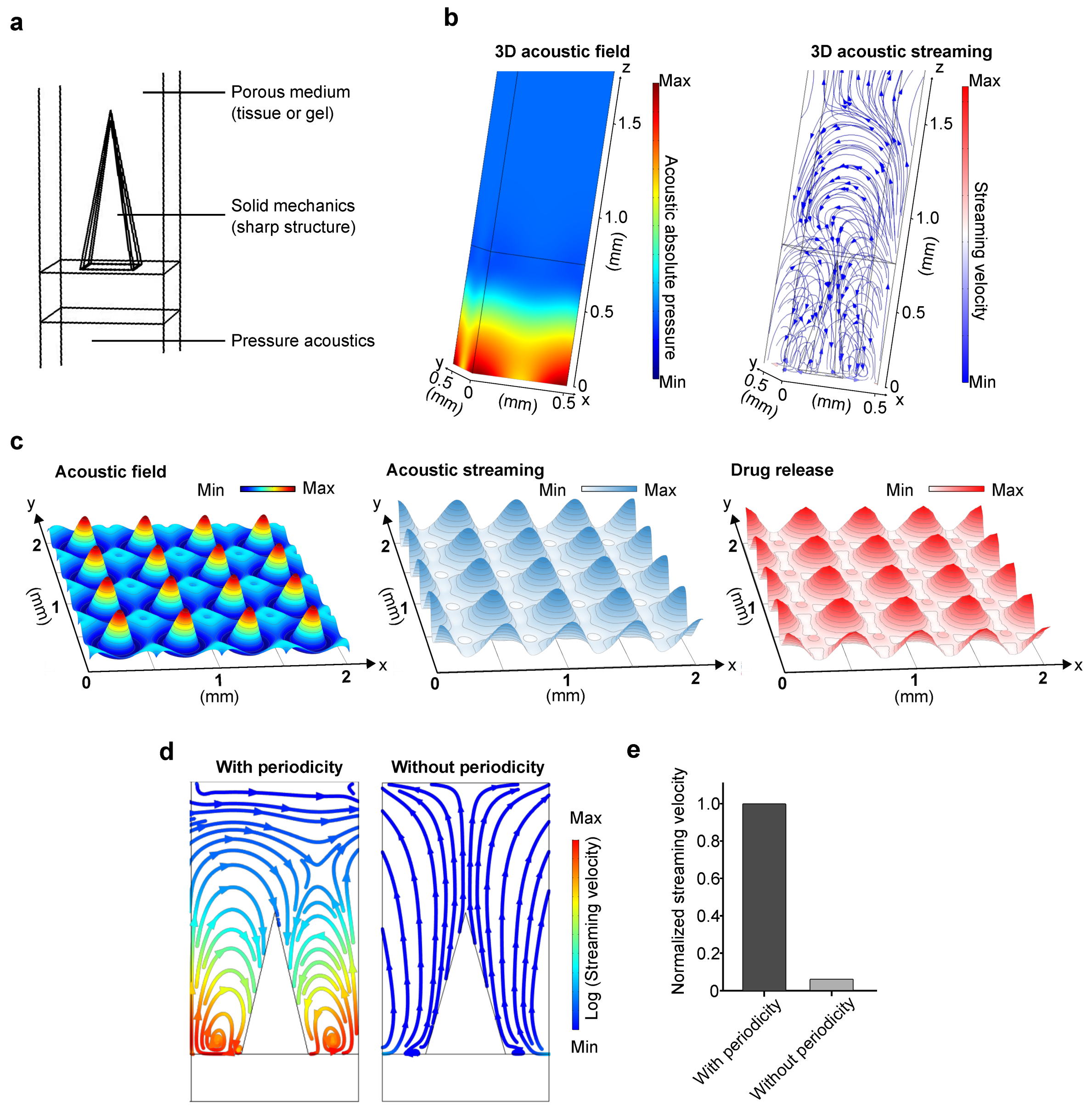

Fig.S2 Simulation of the acoustic field, acoustic streaming, dye release, and periodicity driven by acoustic metamaterials. (**a**) Schematic of a computational domain for simulating a unit of periodic metamaterial structures. (**b**) 3D simulated distributions of the acoustic and streaming fields mediated by acoustic metamaterial structures. (**c**) Acoustic metamaterial mediated 3D simulated distributions of acoustic field, acoustic streaming, and released dye above the sharp metamaterial structures. (**d**) 3D simulated distributions of the acoustic streaming fields mediated by a metamaterial structure with or without periodicity. (**e**) The acoustic streaming velocity driven by a metamaterial structure with or without periodicity, indicating the power of acoustic metamaterials.

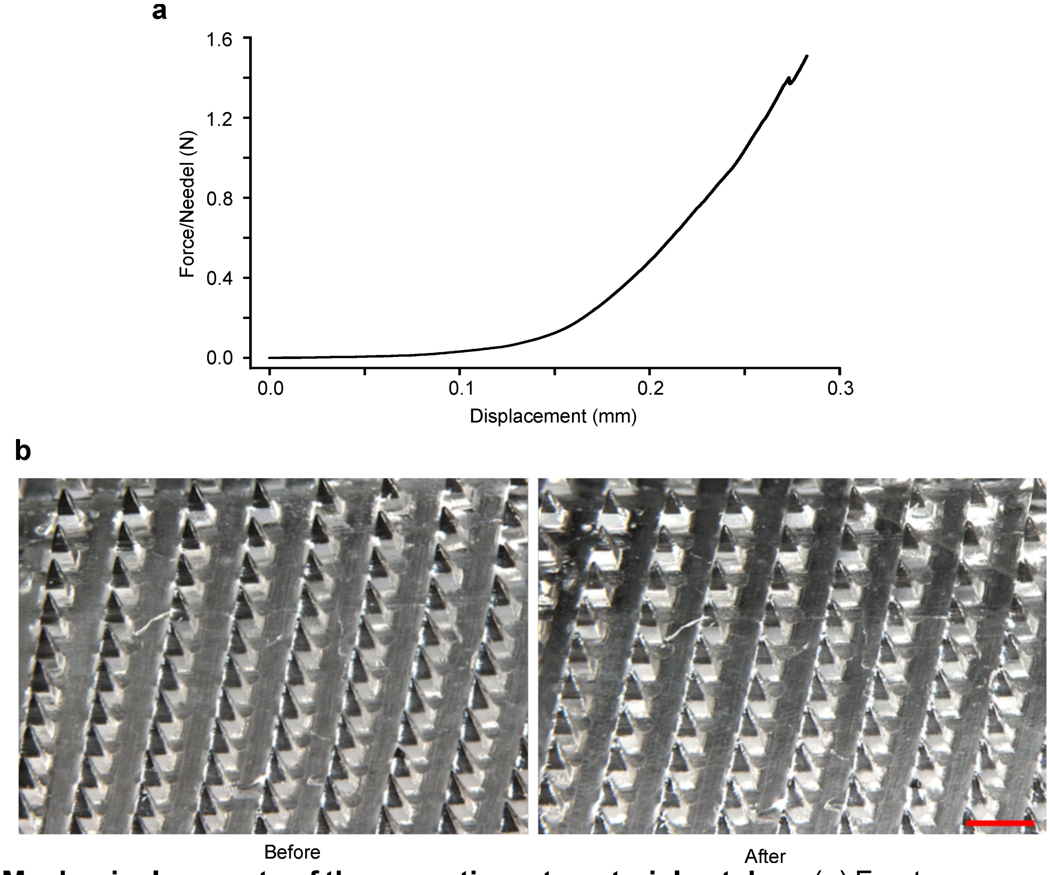

Fig.S3 Mechanical property of the acoustic metamaterial patches. (**a**) Fracture occurred upon applying ~1.4 N in metamaterial structures. (**b**) Images of metamaterial structures of an acoustic metamaterial patch before and after injection into the animal skin. The experiment was repeated 3 times independently with similar results. (Scale bar: 500 μm)

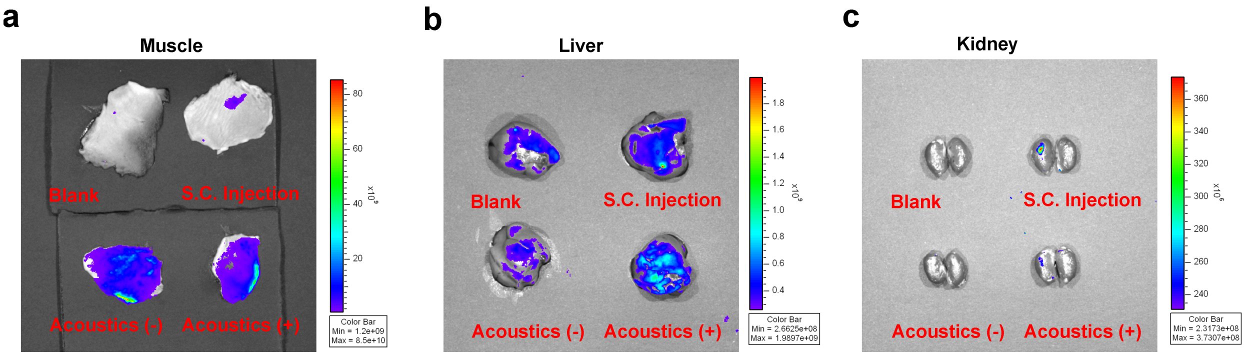

Fig.S4 *In vivo* dye distribution mediated by acoustic metamaterial patches and other methods. Representative images of harvested mouse muscle (**a**), liver (**b**), kidney (**c**) tissues after dye delivery mediated via an acoustic metamaterial patch with acoustic stimulation (Acoustics (+)), without acoustic stimulation (Acoustics (-)), via subcutaneous injection (S.C. injection), and without any treatment (Blank).

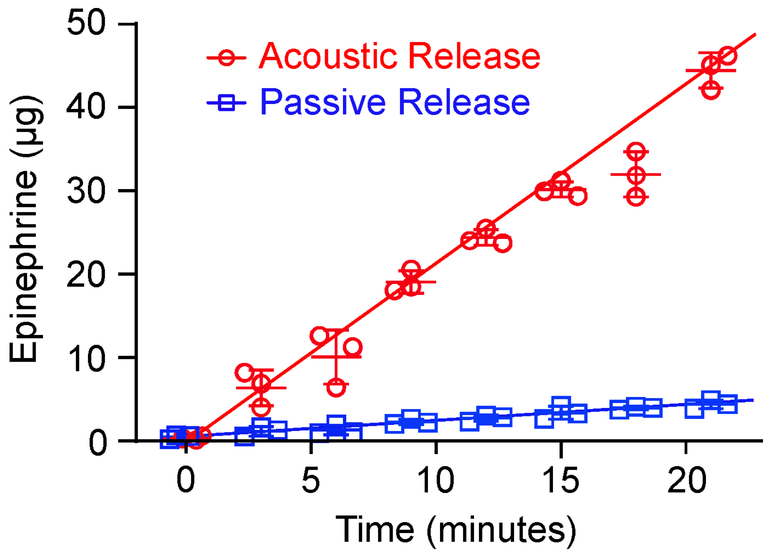

Fig.S5 Controlled release of epinephrine via acoustic metamaterial patches.
(mean ± s.e.m., n = 3 independent experiments)

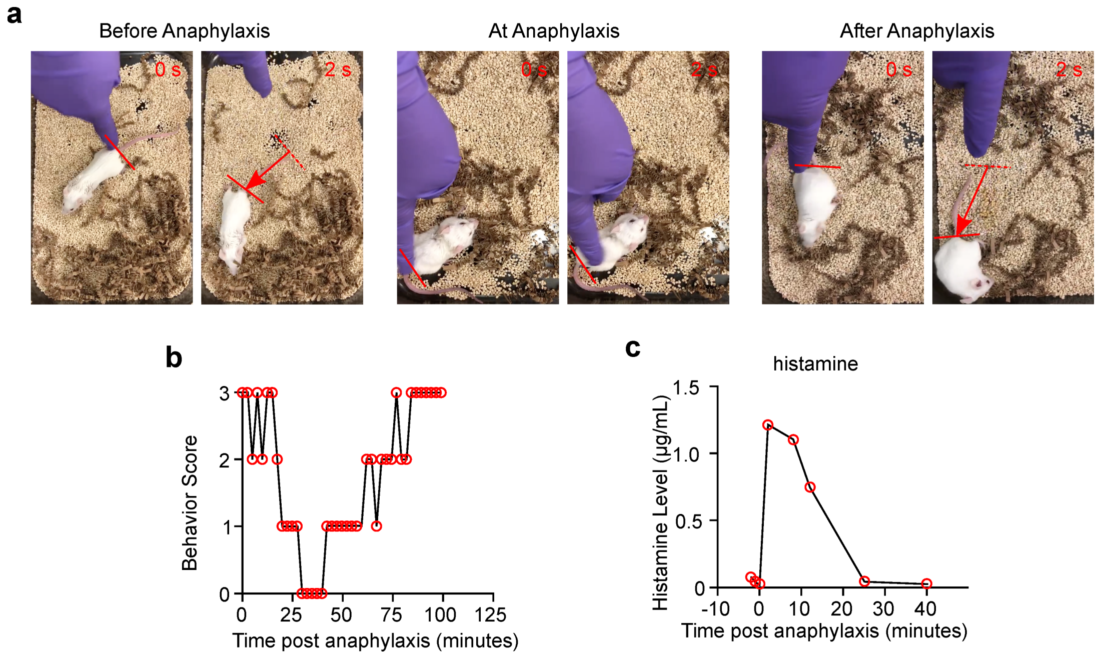

Fig.S6 Characterization of a mouse model of anaphylaxis. (**a**) Representative behaviors of mice before, during, and after anaphylaxis. (**b**) The behavior score of a mouse before, during, and after anaphylaxis. (**c**) Serum histamine elevation of a mouse during anaphylaxis.

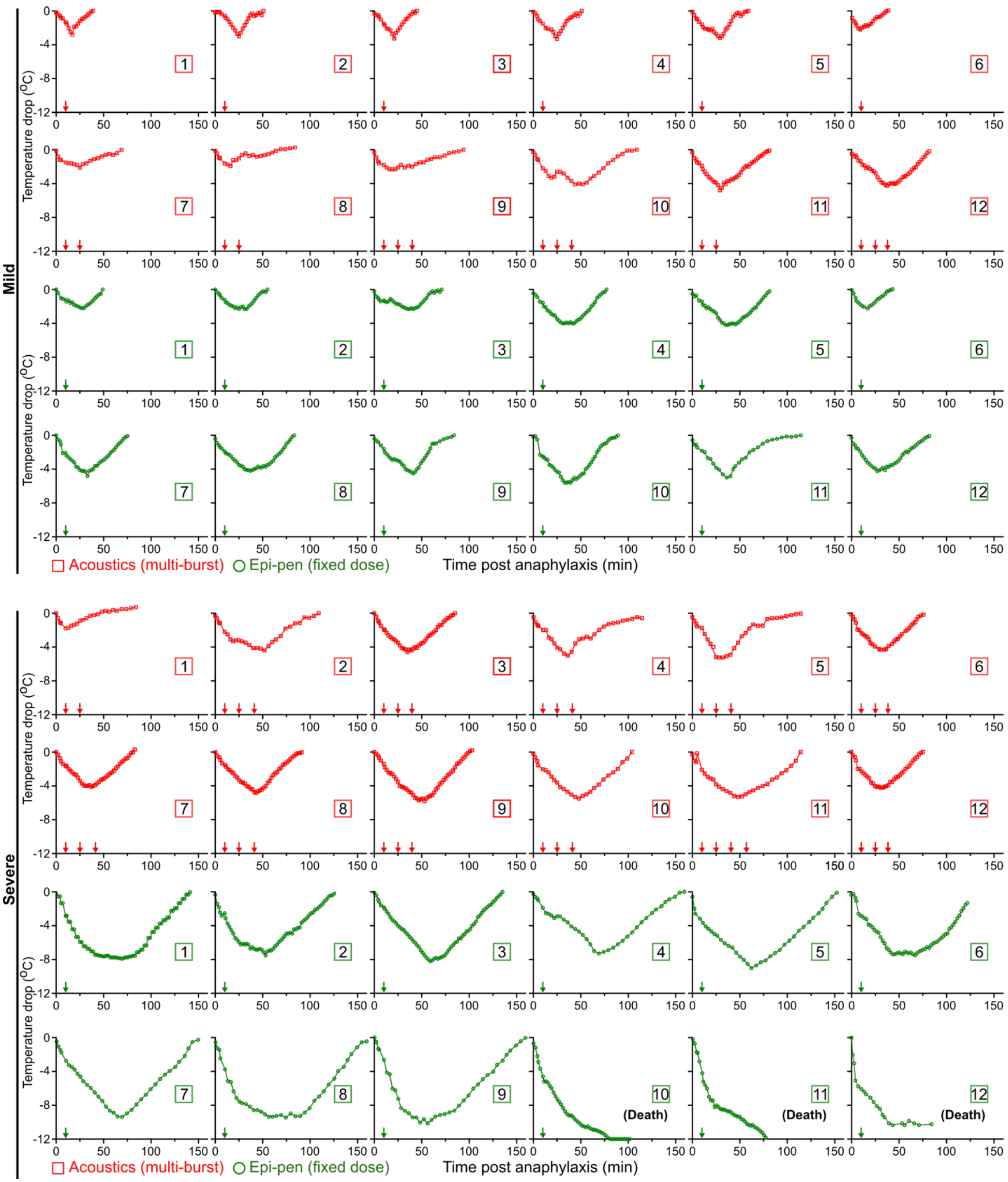

Fig.S7 Body temperature variation of anaphylactic mice subjected to different treatments. Dynamic body temperature change in mice with two levels (mild or severe) of anaphylaxis receiving a multi-burst delivery of epinephrine via acoustic metamaterial patches or the fixed dosage delivery of epinephrine via ‘epi-pen strategy’. The arrows indicate the injection of epinephrine (n= 12 mice per group, independent experiments).

Supplementary References

1. Coussy, O. Poromechanics. (John Wiley & Sons Ltd, Chichester; 2004).
2. Hamilton, M.F. & Blackstock, D.T. Nonlinear acoustics. (Academic Press, San Diego, CA; 1998).
3. Dafermos, C.M. et al. Hyperbolic conservation laws in continuum physics, Vol. 3. (Springer, 2005).
