# Supplementary material for: Acoustic metamaterials-driven transdermal drug delivery for rapid and on-demand management of acute disease
Source: Nat Commun. 2023 Feb 16;14:869. doi: 10.1038/s41467-023-36581-2 (PMC9935629; doi:10.1038/s41467-023-36581-2)
Supplement: Supplementary file 2 — Description of Additional Supplementary Files [file 41467_2023_36581_MOESM2_ESM.pdf]

Description of Additional Supplementary Files

File name: Supplementary Movie 1

Description: Simulated 3D acoustic streaming mediated by acoustic materials.

File name: Supplementary Movie 2

Description: Experimental 3D acoustic streaming mediated by acoustic materials.

File name: Supplementary Movie 3

Description: Simulated dye release process via acoustic materials.

File name: Supplementary Movie 4

Description: Experimental dye release process via acoustic materials.

File name: Supplementary Movie 5

Description: A free-moving mouse with an acoustic metamaterial patch device.
