# Supplementary material for: Acoustic metamaterials-driven transdermal drug delivery for rapid and on-demand management of acute disease
Source: Nat Commun. 2023 Feb 16;14:869. doi: 10.1038/s41467-023-36581-2 (PMC9935629; doi:10.1038/s41467-023-36581-2)
Supplement: Supplementary file 8 — Reporting Summary [file 41467_2023_36581_MOESM8_ESM.pdf]

Corresponding author(s): Feng Guo

Last updated by author(s): Dec 29, 2022

Reporting Summary

Nature Portfolio wishes to improve the reproducibility of the work that we publish. This form provides structure for consistency and transparency in reporting. For further information on Nature Portfolio policies, see our Editorial Policies and the Editorial Policy Checklist.

Statistics

For all statistical analyses, confirm that the following items are present in the figure legend, table legend, main text, or Methods section.

n/a	Confirmed	
☐	☒	The exact sample size (*n*) for each experimental group/condition, given as a discrete number and unit of measurement
☐	☒	A statement on whether measurements were taken from distinct samples or whether the same sample was measured repeatedly
☐	☒	The statistical test(s) used AND whether they are one- or two-sided<br>*Only common tests should be described solely by name; describe more complex techniques in the Methods section.*
☒	☐	A description of all covariates tested
☐	☒	A description of any assumptions or corrections, such as tests of normality and adjustment for multiple comparisons
☐	☒	A full description of the statistical parameters including central tendency (e.g. means) or other basic estimates (e.g. regression coefficient) AND variation (e.g. standard deviation) or associated estimates of uncertainty (e.g. confidence intervals)
☐	☒	For null hypothesis testing, the test statistic (e.g. *F*, *t*, *r*) with confidence intervals, effect sizes, degrees of freedom and *P* value noted<br>*Give P values as exact values whenever suitable.*
☒	☐	For Bayesian analysis, information on the choice of priors and Markov chain Monte Carlo settings
☐	☒	For hierarchical and complex designs, identification of the appropriate level for tests and full reporting of outcomes
☒	☐	Estimates of effect sizes (e.g. Cohen's *d*, Pearson's *r*), indicating how they were calculated

Our web collection on statistics for biologists contains articles on many of the points above.

Software and code

Policy information about availability of computer code

Data collection	The data were collected with various instrumentations and equipment such as microscope (Olympus, IX83), in vivo imaging system (IVIS Spectrum, Perkin Elmer), etc.
Data analysis	Origin 9.1, Graph Pad Prism 9, Image J, COMOSOl 5.3a

For manuscripts utilizing custom algorithms or software that are central to the research but not yet described in published literature, software must be made available to editors and reviewers. We strongly encourage code deposition in a community repository (e.g. GitHub). See the Nature Portfolio guidelines for submitting code & software for further information.

Data

Policy information about availability of data

All manuscripts must include a data availability statement. This statement should provide the following information, where applicable:

- Accession codes, unique identifiers, or web links for publicly available datasets
- A description of any restrictions on data availability
- For clinical datasets or third party data, please ensure that the statement adheres to our policy

The authors declare that all the relevant data supporting the findings of this study are available within the article and the Supplementary Information and Supplementary Movices.

Human research participants

Policy information about studies involving human research participants and Sex and Gender in Research.

Reporting on sex and gender	n/a
Population characteristics	n/a
Recruitment	n/a
Ethics oversight	n/a

Note that full information on the approval of the study protocol must also be provided in the manuscript.

Field-specific reporting

Please select the one below that is the best fit for your research. If you are not sure, read the appropriate sections before making your selection.

☒ Life sciences ☐ Behavioural & social sciences ☐ Ecological, evolutionary & environmental sciences

For a reference copy of the document with all sections, see nature.com/documents/nr-reporting-summary-flat.pdf

Life sciences study design

All studies must disclose on these points even when the disclosure is negative.

Sample size	We have adopted at least n=3 biological replicates to calculate the statistical value of each analysis and the exact sample sizes and statistical data for each experiment are reported in the figure legends (EMBO Rep. 2012, 13(4): 291–296).
Data exclusions	No data is excluded.
Replication	All the experiments are triplicated. All attempts at replication were successful and the exact numbers are indicated in the figure legends or table for all experiments.
Randomization	The animals were assorted randomly to cages when received from the vendor.
Blinding	The investigators were blinded to group allocation during data collection and analysis.

Reporting for specific materials, systems and methods

We require information from authors about some types of materials, experimental systems and methods used in many studies. Here, indicate whether each material, system or method listed is relevant to your study. If you are not sure if a list item applies to your research, read the appropriate section before selecting a response.

Materials & experimental systems

n/a	Involved in the study	
☐	☒	Antibodies
☒	☐	Eukaryotic cell lines
☒	☐	Palaeontology and archaeology
☐	☒	Animals and other organisms
☒	☐	Clinical data
☒	☐	Dual use research of concern

Methods

n/a	Involved in the study	
☒	☐	ChIP-seq
☒	☐	Flow cytometry
☒	☐	MRI-based neuroimaging

Antibodies

Antibodies used	monoclonal mouse anti-DNP IgE antibody (D8406, Clone SPE-7, Sigma)
Validation	Th antibody used in our experiments is commercially available and has been validated by the manufacturer. All validation statements can be found on the respective antibody website: https://www.sigmaaldrich.com/US/en/product/sigma/d8406

Animals and other research organisms

Policy information about studies involving animals; ARRIVE guidelines recommended for reporting animal research, and Sex and Gender in Research

Laboratory animals	C57BL/6J mice, BALB/c mice (6-12 weeks of age, Envigo), and all of the mice were housed as five mice in a cage under conventional conditions (12 h light/dark cycle at 22 ºC).
Wild animals	No wild animal has been used here.
Reporting on sex	We used only female mice to validate our patch devices in our experiments. Anaphylaxis has been reported with higher frequency in adult females than in males (Cur Opin Allergy Clin Immunol. 2019(5):417-424). Thus, we chose only female mice to validate our patch device and demonstrate effective anaphylaxis reversal using our method.
Field-collected samples	The study didn't involve samples from the field.
Ethics oversight	All experiments were conducted following the animal guidelines of the institutional animal care and use (IACUC) committee of Indiana University as approved under protocol # 19-006.

Note that full information on the approval of the study protocol must also be provided in the manuscript.
